# Genetic diversification patterns in swine influenza A virus (H1N2) in vaccinated and nonvaccinated animals

**DOI:** 10.3389/fcimb.2023.1258321

**Published:** 2023-09-15

**Authors:** Álvaro López-Valiñas, Marta Valle, Marta Pérez, Ayub Darji, Chiara Chiapponi, Llilianne Ganges, Joaquim. Segalés, José I. Núñez

**Affiliations:** ^1^ IRTA, Programa de Sanitat Animal, Centre de Recerca en Sanitat Animal (CReSA), Bellaterra, Barcelona, Spain; ^2^ Unitat Mixta d’Investigació IRTA-UAB en Sanitat Animal, Centre de Recerca en Sanitat Animal (CReSA), Universitat Autònoma de Barcelona (UAB), Barcelona, Spain; ^3^ WOAH Collaborating Centre for the Research and Control of Emerging and Re-Emerging Swine Diseases in Europe (IRTA-CReSA), Barcelona, Spain; ^4^ WOAH Reference Laboratory for Swine Influenza, Istituto Zooprofilattico Sperimentale della Lombardia ed Emilia-Romagna, Brescia, Italy; ^5^ WOAH Reference Laboratory for Classical Swine Fever, IRTA-CReSA, Barcelona, Spain; ^6^ Departament de Sanitat i Anatomia Animals, Universitat Autònoma de Barcelona, Barcelona, Spain

**Keywords:** swine influenza virus, viral evolution, vaccination, NGS, quasispecies

## Abstract

Influenza A viruses (IAVs) are characterized by having a segmented genome, low proofreading polymerases, and a wide host range. Consequently, IAVs are constantly evolving in nature causing a threat to animal and human health. In 2009 a new human pandemic IAV strain arose in Mexico because of a reassortment between two strains previously circulating in pigs; Eurasian “avian-like” (EA) swine H1N1 and “human-like” H1N2, highlighting the importance of swine as adaptation host of avian to human IAVs. Nowadays, although of limited use, a trivalent vaccine, which include in its formulation H1N1, H3N2, and, H1N2 swine IAV (SIAV) subtypes, is one of the most applied strategies to reduce SIAV circulation in farms. Protection provided by vaccines is not complete, allowing virus circulation, potentially favoring viral evolution. The evolutionary dynamics of SIAV quasispecies were studied in samples collected at different times from 8 vaccinated and 8 nonvaccinated pigs, challenged with H1N2 SIAV. In total, 32 SIAV genomes were sequenced by next-generation sequencing, and subsequent variant-calling genomic analysis was carried out. Herein, a total of 364 *de novo* single nucleotide variants (SNV) were found along all genetic segments in both experimental groups. The nonsynonymous substitutions proportion found was greater in vaccinated animals suggesting that H1N2 SIAV was under positive selection in this scenario. The impact of each substitution with an allele frequency greater than 5% was hypothesized according to previous literature, particularly in the surface glycoproteins hemagglutinin and neuraminidase. The H1N2 SIAV quasispecies evolution capacity was evidenced, observing different evolutionary trends in vaccinated and nonvaccinated animals.

## 1 Introduction

Influenza A viruses (IAVs) are worldwide distributed pathogens with a very wide host range, being able to infect many birds and mammal species, and with increasing zoonotic potential (Webster et al., 1992). The IAV genome is composed of 8 single-stranded viral RNA segments of negative polarity, with a total length of 13.6 kb (Zhou et al., 2009). The genomic segments have different sizes, ordered from largest to smallest size as follows: the polymerase basic 2 (PB2), polymerase basic 1 (PB1), polymerase acid (PA), surface glycoproteins hemagglutinin (HA), nucleoprotein (NP), neuraminidase (NA), matrix segment (M), and non-structural (NS) (Breen et al., 2016). Each segment codes for at least one protein with the same name, except M and NS segments that also code for the ion channel protein (M2) and the nuclear export protein (NS2/NEP), respectively, by alternative RNA splicing (Lamb et al., 1980; Lamb et al., 1981). Furthermore, there are mRNAs with open reading frames that code for accessory viral proteins recently identified, such as the polymerase basic 1 frame 2 (PB1-F2), and the PA-X proteins (Wise et al., 2009; Jagger et al., 2012; Hao et al., 2020).

The evolution of IAVs, such as other RNA viruses, is described under the quasispecies theory, where a viral population is composed of a spectrum or cloud of mutants (Eigen, 1971; Eigen and Schuster, 1977; Bull et al., 2005; Lauring and Andino, 2010; Schuster, 2016; Domingo and Perales, 2019). This implies an adaptive advantage for the virus because of the phenotypic variants reservoir that could be beneficial for virus adaptability in pressure events, such as immune escape mutants and drug resistance (Domingo et al., 2012; Schuster, 2016). There are three molecular mechanisms that provide genetic variability to the IAV genomes, influencing its evolution: point mutations, genomic reassortments, and recombination, although the last phenomenon does not seem to play an important role during IAV evolution (Chare et al., 2003; Krammer et al., 2018).

The addition of point mutations in IAVs is due to the low rate of fidelity of their polymerases. Consequently, RNA viruses can add and accumulate a lot of point mutations in their genomes during their replication. The IAVs mutation rate is high, on the order between 10^-3^ and 10 ^-4^ substitution per gen per generation, allowing rapid and progresSIAVe evolution of the virus (Ahlquist, 2002). IAV genome regions such as the epitope regions of the surface glycoproteins HA and NA are constantly under positive evolutionary pressure since they are the main target for the host immune system. Over time, these regions can evolve until they are no longer recognized by the previous immunity of the host population, in case of existing, therefore generating escape variants (Shao et al., 2017). This phenomenon is known as antigenic drift, which can generate new viral subtypes (Nelson and Holmes, 2007; Russell et al., 2008). The accumulation of point mutations could influence on IAVs host range, virulence, and antiviral resistance (Domingo et al., 2012; Shao et al., 2017).

Therefore, due to the pandemic risk posed by swine IAVs (SIAVs), deeper virus genetic evolution mechanism understanding and constant surveillance studies in the field are required. Currently, “avian-like” swine H1N1, “human-like” swine H1N2, “human-like” reassortment swine H3N2, and A(H1N1) pdm09 virus, are the most circulating subtypes among the pig population (Simon-Grifé et al., 2011; Torremorell et al., 2012; Kyriakis et al., 2013; Simon et al., 2014; Li and Robertson, 2021). Nowadays, vaccination against SIAV, although limited, is the most effective and used strategy to control SIAV in the swine population, being administered to approximately 10-20% of the European pig population (Reeth et al., 2016). One of the most used vaccines against SIAV is the trivalent one, which includes in its formulation inactivated strains of the H1N1, H3N2, and H1N2 SIAV subtypes. This vaccine reduces both, disease signs and viral shedding. However, due to the genomic distance between farm circulating and vaccine formulation strains, the protection generated during vaccination is not complete, allowing virus circulation, potentially favoring viral evolution (Thacker and Janke, 2008; Ma and Richt, 2010). In previous studies, the viral evolution patterns of the SIAV H1N1 and H3N2 subtypes were analyzed in vaccinated and nonvaccinated pigs, showing different evolutionary patterns in both experimental groups (López-Valiñas et al., 2021; López-Valiñas et al., 2022; López-Valiñas et al., 2023). However, the evolutionary pressure generated by the trivalent vaccine on SIAV H1N2 subtype is still poorly studied.

Considering this background, the present work aimed to study the evolutionary dynamics of SIAV H1N2 quasispecies under immunity pressure and the identification of *de novo* nonsynonymous substitutions that may have an impact on viral fitness and host immune system evasion. Therefore, 32 whole SIAV genomes recovered at different time points from challenged vaccinated and nonvaccinated pigs were determined by next-generation sequencing (NGS), showing different evolutionary patterns between both experimental groups.

## 2 Materials and methods

### 2.1 Cells, viruses, and vaccine

Madin-Darby Canine Kidney (MDCK, ATCC CCL-34) cells were used for viral production and titration. Dulbecco’s Modified Eagle Medium (DMEM) supplemented with foetal bovine serum (FBS) (10%), L-glutamine (1%) and penicillin/streptomycin (1%) was used for cell culture in an incubator at 37°C with 5% CO_2_ atmosphere.

The strain (A/Swine/Spain/80598LP4/2007(H1N2)) used in this study was first propagated in MDCK monolayer cell culture at a multiplicity of infection (MOI) of 0.01. Virus cell infection was promoted applying 10 µg/mL of porcine trypsin (Sigma-Aldrich, Madrid, Spain). After 48 hours, infected cells were harvested. For viral titration, the 50% tissue culture infection dose (TCID_50_) was calculated carrying out a serial dilution in MDCK cells (Reed and Muench, 1938).

In the present study, a commercial vaccine against SIAV (RESPIPORC FLU3, Ceva^®^, Libourne, France), which includes the H1N2 (Bakum/1832/2000), H1N1 Haselünne/IDT2617/2003), and H3N2 (Bakum/IDT1769/2003) inactivated strains in its formulation, was used.

### 2.2 Experiment design and samples collection

Sixteen 5-week-old naïve domestic pigs were used. Pigs were randomly distributed in two groups (A and B) of 8 animals each, located in different boxes at the animal biosafety level 3 (BSL3) facilities at IRTA-CReSA (Bellaterra, Spain). The animals spent an acclimation period of 1 week. After that, pigs from group A were vaccinated according to manufacturer’s instruction, with administration of 2 mL intramuscularly in the neck. Animals from group B, received in parallel 2 mL of phosphate buffered saline (PBS) administrated as previously mentioned. A second vaccination dose were carried out 21 days post-vaccination (21 dpv), where animals from group A and B received the commercial vaccine and PBS, respectively.

All animals were challenged with (A/Swine/Spain/80598LP4/2007(H1N2) strain, three weeks after the second vaccination dose (42 dpv). The virus was inoculated thorough two different administration routes, intranasal and endotracheal. The final viral concentration inoculated was 10^7^ TCID_50_ in a final volume of 2 mL for the intranasal and 5 mL for the endotracheal inoculation, as previously described (López-Valiñas et al., 2021). After challenged, animals were serially euthanized, 3 from each group 2 days post inoculation (2 dpi), 3 per group at 5dpi, and the remaining 4 pigs by the end of the *in vivo* experiment (9 dpi).

Nasal swab samples were collected at each immunization, at challenge, and daily post-challenge until the euthanasia day, for SIAV detection and genome sequencing. Blood samples were collected at each immunization, challenge, and necropsy day for antibody detection against SIAV. At necropsy, lung, nasal turbinate (NT) and broncho-alveolar lavage fluid (BALF) were collected and stored at -80°C, for SIAV detection and NGS. Furthermore, a lung extra sample was stored in formalin for pathological analysis.

After challenge, rectal temperature, clinical signs (Galindo-Cardiel et al., 2013)and animal behaviour were daily monitored. Procedures were approved by the animal ethics committee from the *Generalitat de Catalunya*, under the project number 10442, following the Spanish and European regulations.

### 2.3 Evaluation of the humoral response against SIAV

In serum samples collected, the antibody levels against influenza NP were calculated using ID Screen^®^—influenza A Antibody Competition ELISA kit (ID VET, Grabels, France), following manufacturer instructions. Inhibition percentages lower than 45% indicate positive sera, greater than 50% negative ones, and percentages between both values indicate doubtful results according to kit instructions.

### 2.4 Pathological analyses and immunohistochemistry in lung

The percentage of the lung-affected area of each necropsied animal was measured with ImageJ^®^ software, as previously described (Sibila et al., 2014).

A sample of each lung showing gross lesion was fixed by immersion in 10% buffered formalin. After that, each lung section was dehydrated, and embedded in paraffin wax. For light microscopy examination, hematoxylin-eosin (HE) staining and immunohistochemistry (IHC) were done separately in sections of tissue with a thickness of 3-5 μm (Busquets et al., 2010; Sisteré-Oró et al., 2019). For IHC, the two-step polymer method (Envision TM) was performed using a primary and secondary antibody (Sabattini et al., 1998). For the evaluation of the lung histopathological findings and the amount of immunoreactivity on HE and IHC slides, respectively, previously described semi-quantitative scoring methods were used (Detmer et al., 2013).

### 2.5 SIAV RNA detection, whole genome amplification, and sequencing

The MagAttract 96 Cador Pathogen kit ^®^ (Qiagen, Düsseldorf, Germany) was used to extract RNA from nasal swabs, BALF, lung, and NT samples, following the manufacturer’s instructions. Lung and NT samples were previously homogenized with TissueLyser II (Qiagen, Düsseldorf, Germany) in brain heart infusion medium (10% weight/volume).

SIAV RNA detection and quantification were performed on each sample using an RT-qPCR assay for IAV in the Fast7500 equipment (Applied Biosystem) (Spackman et al., 2002). Samples that showed fluorescence were considered positive and the cycle threshold (Ct) value parameter obtained was used for SIAV quantification (Spackman et al., 2002; López-Valiñas et al., 2021).

The inoculum used on the challenge and all BALF and nasal swab samples with a Ct value lower than 35 in RT-qPCR assay were proposed for whole SIAV genome sequencing (Zhou et al., 2009; López-Valiñas et al., 2021).

First, a whole SIAV genome amplification was performed. The PCR reaction mix conditions were as follows: forward MBTuni-12 and reverse MBTuni-13 primers both at 0.2 μM (0.5 μL each), SuperScript^®^ III One-Step RT-PCR System with Platinum™ Taq High Fidelity DNA Polymerase (Thermo Fisher Scientific, Waltham, MA, USA) (0.5 μL), reaction mix included in the kit (12.5 μL), RNasa free water (8.5 μL) and SIAV RNA from each sample(2.5 μL) (Zhou et al., 2009). Furthermore, to enhance the biggest SIAV segments amplification another RT-PCR was carried parallelly out, replacing MBTuni12 primer with MBTuni12G (Lycett et al., 2012). The rest of the RT-PCR conditions were maintained. All amplified samples showing a whole genome amplification profile by agarose gel electrophoresis were selected for sequencing (Zhou et al., 2009).

Illumina technology was used for whole genome SIAV sequencing. Nextera-XT DNA Library Prep protocol (Illumina^®^, San Diego, CA, USA) was followed to obtain a multiplexed sequencing library per sample. Miseq Reagent Kit v2 in a 150-cycle paired-end run on Miseq Instrument was used.

### 2.6 Bioinformatic workflow for quasispecies genomic and evolutionary analysis

Herein, a bioinformatics workflow for aligning Illumina reads against a known reference and variant calling was developed in the bash programming language. In this pipeline, tools widely used for viral genome analysis were applicated. (Sobel Leonard et al., 2016; Borges et al., 2018; Cacciabue et al., 2020).

First, FastQC (v 0.11.8) (Andrews, 2010) software was used for illumina reads quality checking. Low-quality reads (Phread < 30) were trimmed by Trimmomatic (v0.39) (Bolger et al., 2014). Inoculum reads were first aligned against the reference genome H1N2 sequence (HF674912-19) (Baratelli et al., 2014), as explained below. Subsequently, the inoculum consensus sequence was generated using the consensus option from Bcftools (v.1.9) (Danecek et al., 2021). For the subsequent variant calling and genomic analysis, all sequenced samples were mapped against the inoculum consensus sequence.

For reads mapping the Burrows-Wheeler alignment (BWA) tool mem function (v0.7.17) was used (Li and Durbin, 2009). After that, unmapped and low-quality mapped (<30) reads were removed using Samtools (v.0.39) (Danecek et al., 2021). Moreover, the Picard “MArkDuplicatesSpark” and “BaseRecalibrator” algorithms included in GATK4 (v4.1) were used for both; PCR duplicates removal and reads recalibration. The depth of reading of samples on each genomic segment was calculated by “-depth” option included on Samtools (v1.9). The coverage and depth plots were performed with the ggplot2 library in Rstudio (RStudio, 2021; Wickham, 2016).

All variants found along the SIAV genome from sequenced samples in comparison with the inoculum consensus sequence were noted using LoFreq software with default parameters (Wilm et al., 2012). After that, the effect on each variant detected was predicted by SnpEff software (v.4.3) (Cingolani et al., 2012). For this purpose, a database has previously been built using as a reference a prior annotated H1N2 genome (Baratelli et al., 2014) using “build–gtf22” function included in SnpEff. For single nucleotide variant (SNV) calling the following requirement were considered; p value < 0.01, 100 reads of depth, and 10 reads of alternative base count (López-Valiñas et al., 2021). Finally, the nucleotide diversity (π) was calculated by using SNPGenie software (Nelson et al., 2015).

### 2.7 Protein structure representation

For the Lolliplot protein representation, the trackViewer package from Bioconductor (Ou and Zhu, 2019) was used. In addition, the HA and NA 3D protein was represented by the PyMOL Molecular Graphics System (v.4.6). The functional domains of all protein representations were delimited according to previous literature (Sriwilaijaroen and Suzuki, 2012; Hu et al., 2014; McAuley et al., 2019). In the protein structure representations, the nonsynonymous substitutions found with an allele frequency greater than 5% were pointed out.

### 2.8 Statistical analysis

T-test statistical analysis was herein used to determine significant differences between the average values of % competition ELISA, pig rectal temperatures, RT-qPCR Ct values, and % of lung affected area, in vaccinated and nonvaccinated animals at different time points. Before that, all variable distributions and means variances were studied using Shapiro and Levene tests, respectively. Paired t-test was also applied to observe increases in rectal temperatures within the same group on different days. The differences between synonymous and nonsynonymous proportions at different allele frequencies per group were analyzed by applying the Chi-squared test and the subsequent Bonferroni correction. To compare π means, an analysis of variance (ANOVA) and a *post-hoc* Kruskal-Wallis test by rank were applied. All statistical analyses were carried out using R (RStudio, 2021).

## 3 Results

### 3.1 Humoral response, viral replication, and pig rectal temperature kinetics

**Figure 1:**
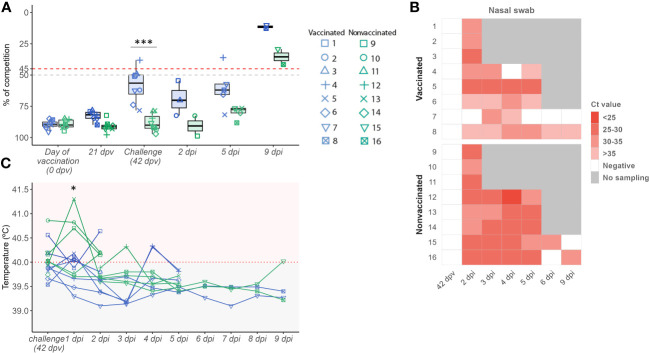
Evaluation of the vaccine effect through humoral response, viral replication, and pig rectal temperature profile. **(A)** SIAV NP antibody titres boxplot. The percentage of competition is expressed in the ordinate axis. Boxplot whiskers show quartile variability. Values below 50% (grey line) are considered negative, while values above 45% (red line) are considered positive. Values between both lines are considered doubtful. **(B)** SIAV detection in nasal swab samples heatmap. In the y-axis, each ID animal number from both experimental groups is indicated. The heat map shows cells with different red intensities, from the darkest to the lightest, indicating a range of viral loads from higher to lower, respectively depending on the Ct value obtained per nasal swab sample. White cells represent SIAV negative samples (Ct values > 40) meanwhile, grey ones indicate no sample was collected at that time point. **(C)** Pig rectal temperature kinetics after SIAV challenge. The temperature measured in °C is represented in the ordinate axis. Horizontal lines represent the temperature profile of each pig over time. Pigs with values above 40°C were considered to have fever. In all figures, the sampling time is indicated in the abscissa axis. Vaccinated, and nonvaccinated animals are represented in blue and green dots, boxplots, and lines, respectively. Besides, the dot shape and number indicate each animal ID. * p < 0.05 and *** p < 0.0001. Paired t-test was used for statistical analysis.

At challenge day, the NP antibody levels mean detected in sera samples from vaccinated animals (58.14 ± 13.54)) were higher than those of nonvaccinated ones (88.16 ± 6.89), being this difference significant (*t-test;p = 0.00000663*) (**Figure 1A**). At this time point, vaccinated animals No. 3 and 4 were considered positive, No. 1 and 8 doubtful, and the remaining ones negative. On the other hand, all sera samples from nonvaccinated animals were considered negative, where any antibody levels detected exceeded the lowest value of vaccinated ones. After the challenge, the SIAV NP ELISA values increased over time, maintaining as average higher values in vaccinated animals.

In the present study, SIAV RNA was detected after the challenge in both experimental groups. On average, viral loads detected along time in nasal swab samples were always higher in nonvaccinated animals reaching significant differences at 4 and 5 dpi (*t-test*; *p=0.00628, and p= 0.02927, respectively*) (**Figure 1B**; **Table 1**). No SIAV was detected on the day of the challenge. Furthermore, SIAV genome was detected in all BALF, lung, and NT samples from both experimental groups at 2 and 5 dpi with no significant differences in Ct values between groups. At 9 dpi, the detected Ct values were close to the detection limit, being negative in all samples collected from vaccinated animal No. 7. SIAV RNA was not detected in NT samples at 9 dpi.

**Table 1:** SIAV RNA detection in nasal swabs, BALF, lung, and NT samples collected from vaccinated and nonvaccinated pigs.

Group	Pig ID	Nasal swab samples							Euthanasia day		Tissue Sample	
		0dpi	2dpi	3dpi	4dpi	5dpi	6dpi	9dpi		BALF	Lung	NT
*Vaccinated Animals*	*1*	*Neg.*	32.6						2 dpi	**24.22**	23.91	25.1
	*2*	*Neg.*	31.5						**29.73**	31.5	27.42
	*3*	*Neg.*	29.15						27.93	19.56	24.58
	*4*	*Neg.*	34.72	32.44	*Neg.*	37.49			5 dpi	**31.12**	31.71	28.41
	*5*	*Neg.*	25.66	**26.41**	**28.72**	**28.76**			**27.67**	23.92	28.35
	*6*	*Neg.*	35.22	38.55	**33.88**	36.59			**26.13**	30.2	32.64
	*7*	*Neg.*	*Neg.*	**33.17**	**36.37**	*Neg.*	*Neg.*	*Neg.*	9 dpi	*Neg.*	*Neg.*	*Neg.*
	*8*	*Neg.*	37.38	35.47	**32.68**	31.56	37.55	37.08	37.86	36.97	*Neg.*
	*mean*		32.32	33.21	32.91	33.60	37.55	37.08		29.24	28.25	27.75
	sd		3.683	4.011	2.762	3.594	0.000	0.000		4.091	5.548	2.642
*Nonvaccinated Animals*	*9*	*Neg.*	29.66						2 dpi	**29.99**	27.44	32.43
	*10*	*Neg.*	32.22						**29.73**	25.22	27.42
	*11*	*Neg.*	28.26						**23.62**	23.15	23.85
	*12*	*Neg.*	29.61	**27.26**	**24.74**	**31.02**			5 dpi	**26.7**	25.3	27.66
	*13*	*Neg.*	32.1	**33.57**	**28.1**	**28.45**			**25.91**	22.38	28.35
	*14*	*Neg.*	30.38	**28.09**	**26.07**	27.65			**24.33**	26.26	26.26
	*15*	*Neg.*	27.11	**25.35**	27.21	**30.08**	33.98	*Neg.*	9 dpi	39.01	36.48	*Neg.*
	*16*	*Neg.*	27.77	**29.18**	**27.55**	**28.01**	*Neg.*	32.97	35.04	*Neg.*	*Neg.*
	*mean*		29.64	28.69	26.73	29.04	33.98	32.97		29.29	26.60	27.66
	sd		1.89	3.07	1.34	1.44				5.39	4.69	2.82

Neg. (negative). Bold numbers indicate sequenced samples.

The table shows the RT-qPCR Ct values obtained per sample. The columns below nasal swab samples indicate the experiment day of collection. The euthanasia day of each pig is also indicated. Means and standard deviation per day, per sample, and per group are indicated.

In total, 42 and 46 out of 51 nasal swabs, BALF and tissue samples analyzed after the challenge tested positive for SIAV in vaccinated and nonvaccinated animals, respectively. In vaccinated animals only 60% of the samples reached Ct values lower than 35, while in nonvaccinated animals this percentage increased until 84% of the total samples analyzed within the group (**Table 1**).

Regarding BALF, lung, and nasal turbinate samples, SIAV RNA was detected in all samples collected at 2 and 5 dpi with ct values lower than 35 (**Figure 1B**; **Table 1**). Notably, in euthanized animals at 9 dpi, SIAV was also detected in BALF and lung samples from animals 8 and 15, and also in BALF from animal 15, all with a ct value greater than 35. SIAV was not detected in NT samples collected at 9 dpi. No significant differences were observed between groups, showing similar mean values

One day after H1N2 SIAV challenge, 4 vaccinated and 6 nonvaccinated animals had fever (**Figure 1C**). On average, higher rectal temperatures were found at 1 dpi in nonvaccinated animals (40.93 ± 0.33) in comparison with vaccinated ones (39.83 ± 0.296), being this difference significant (*t-test; p = 0.0075*) (**Figure 1C**; **Supplementary T** **able S1**). Furthermore, temperatures only increased significantly from challenge day to 1 dpi, and from 1 dpi to 2 dpi, in nonvaccinated animals (*paired t-test; p = 0,044 and p=0.0196, respectively.*) (**Supplementary Table 1**). The remaining days, no significant differences between groups were found, except at 3 dpi where the temperature was also higher in nonvaccinated pigs (*t-test; p = 0.01579*).

### 3.2 Pathological findings

On average, the percentage of lung affected areas was lower in vaccinated animals, although the difference between both groups was not significant (**Table 2**). In vaccinated pigs, the gross pneumonic lesion percentage was lower than 1 in all animals, except in animal No. 1 which percentage reached the highest value among all necropsied animals at 2 dpi (5.12%). On the other hand, in nonvaccinated animals the highest percentage of lung-affected area were recorded at 5 dpi.

**Table 2:** Lung pathology.

Group	Pig ID	Euthanasia day	Lung affected area (%)	Airway histopathological score	Immunoreactivity score
*Vaccinated Animals*	*1*	2 dpi	5.12	3	++
	*2*		0.71	1	+
	*3*		0.50	2	+
	*4*	5 dpi	1.00	2	–
	*5*		0.75	1	+++
	*6*		0.28	1	+++
	*7*	9 dpi	0.00	0.5	–
	*8*		0.21	0.5	–
		*mean*	1.07	1.38	
*Nonvaccinated Animals*	*9*	2 dpi	1.78	2.5	++
	*10*		1.90	2.5	+++
	*11*		0.68	2.5	+++
	*12*	5 dpi	6.78	2.5	+
	*13*		5.14	2.5	+
	*14*		3.19	2.5	+
	*15*	9 dpi	0.64	3	–
	*16*		0.00	0.5	–
		*mean*	2.51	2.31	

Total lung-affected area percentage, airways affected semi-quantitative scoring and amount of immunoreactivity. Immunoreactivity score: absence (-), low (+), scattered (++), moderate (+++), and abundant (++++) amount of immunoreactivity.

Regarding airway histopathological scoring, vaccinated animals showed as average the lowest score. However, vaccinated animal No. 1, together with nonvaccinated animal No. 15 scored the maximum value. Regarding immunohistochemistry labelling, a moderate amount of labelling was detected in vaccinated pigs No. 5, and 6, both necropsied at 5 dpi. By contrast, the highest immunoreactivity score was detected at 2 dpi in nonvaccinated animals. No labelling was detected in animals necropsied at 9 dpi.

Airway histopathological scoring: absence (0), few isolated inflammatory cells (0.5), localized cluster of inflammatory cells (1), several clusters of inflammatory cells in few affected airways (1.5), several clusters of inflammatory cells in several affected airways (2), extenSIAVe clusters of inflammatory cells in several affected airways (2.5), and extenSIAVe inflammation in many airways (3). Immunoreactivity scoring: absence (-), low (+), scattered (++), moderate (+++), and abundant (++++) amount of SIAV antigen.

### 3.3 Whole genome SIAV sequencing of inoculum, nasal swab, and BALF samples

**Figure 2:**
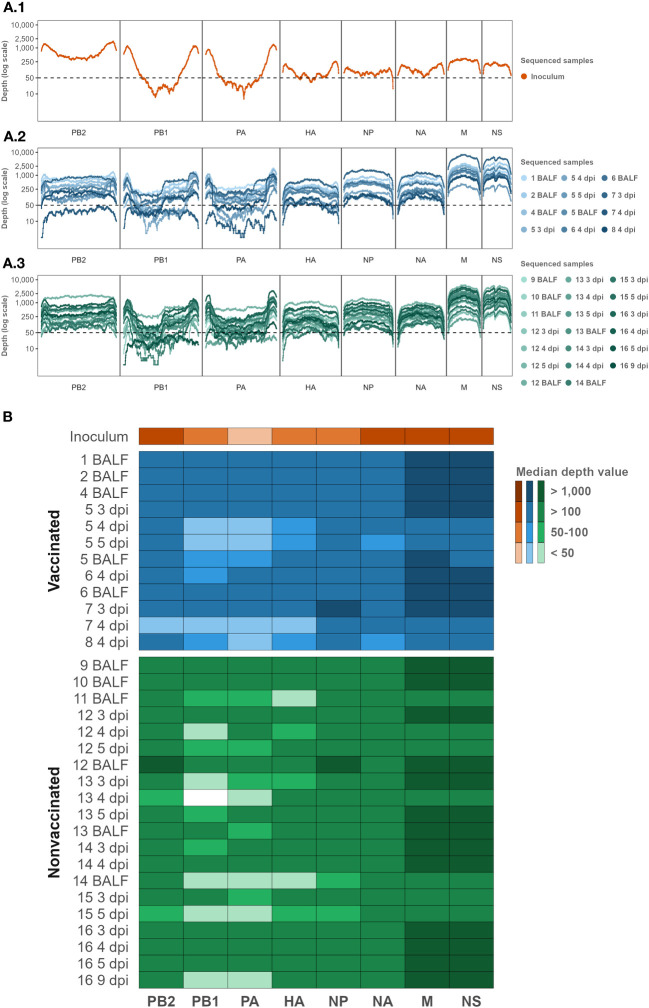
Inoculum, vaccinated, and nonvaccinated NGS sequencing profile samples per genomic segment in terms of depth and coverage. **(A)** The Illumina sequencing profile of SIAV H1N2 is plotted in orange **(A1)**. The Illumina profiles of samples collected from vaccinated and nonvaccinated animals are represented in different tones of blues **(A2)** and greens **(A3)**, respectively. In the abscissa axis, the SIAV genomic segments and each position are represented. In the ordinate axis, the depth of reading is represented in the logarithm scale. **(B)** Median depth value per sample per genomic segment Heat map. The darker colors in the heat map represent higher median depth values; on the contrary, the lighter ones represent a lower value. Inoculum, vaccinated, and nonvaccinated samples are represented in orange, blue, and green respectively. The sequenced samples are represented in the y-axis, where the name indicates the pig ID and the day of sample collection. BALF samples were collected on the day of each pig necropsy. The SIAV genomic segments are indicated on the x-axis.

The SIAV H1N2 strain (A/Swine/Spain/80598LP4/2007(H1N2)) whole genome used in the challenge was determined (**Figure 2A.1**). Regarding samples collected after the challenge, a total of 32 samples were sequenced, 12 samples from vaccinated animals, and 20 from nonvaccinated ones (**Figures 2A.2, 2A.3**; **Table 1**).

After quality control and mapping, a total 1.932.820 Illumina SIAV H1N2 reads were obtained; 32.936, 664.005, and 1.232.879 reads in inoculum, vaccinated, and nonvaccinated sequenced samples. The maximum number of mapped reads obtained per sample were 178.966 in BALF sample collected from nonvaccinated animal No. 12, and 177.727 reads in nasal swab sample collected at 3 dpi from animal No. 7. On the other hand, the minimum number of mapped reads obtained were 14.269, and 14.169 in samples collected from the nonvaccinated animal No. 13 at 4 dpi, and the vaccinated pig No. 5 at 5 dpi, respectively (**Supplementary Table** **S2**).

Regarding the depth and coverage obtained per whole SIAV genome sequenced sample, 88.91% of each genome position sequenced was represented by at least 50 Illumina reads of depth. SIAV H1N2 sequenced positions with more than 50 reads of representation were selected for further variant analysis. In general, the smallest SIAV segments NS and M were sequenced with greater depth representation. By contrast, segments PA and PB1 obtained the lowest depth read representation, followed by the HA segment (**Figure 2B**; **Supplementary Table** **S2**).

### 3.4 Inoculum SNVs and their subsequent detection in pig collected samples

In the inoculum sequence, a total of 10 SNVs were detected; 3 nonsynonymous and 7 synonymous variants. SNVs were only detected in NS1, NA, PA, and PB1 SIAV genes. Most of the genetic substitutions herein reported were found with a low allelic frequency, although 4 of them (NS1(E187K), NA(E156E), PB2(V327V and E399E)) exceeded 10% of allele frequency representation (**Table 3**).

**Table 3:** Single nucleotide variants detected in the inoculum.

	Gene	Nucleotide change		Aminoacidic change		Depth of read	Alt. Base count	Allele frequency	Effect on variant
		position	ref. → alt.	position	ref. → alt.				
Inoculum H1N2	NS1	454	G→A	152	E→K	187	20	10.7	Nsyn
		363	G→T	121	Q→H	229	11	4.8	Nsyn
	NA	1327	A→G	443	I→V	215	18	8.37	Nsyn
		222	G→A	74	E→E	156	29	18.59	Syn
	PA	1779	G→A	593	E→E	128	11	8.59	Syn
	PB2	213	C→T	71	N→N	1085	71	6.54	Syn
		843	G→A	281	L→L	392	15	3.83	Syn
		1209	G→A	403	V→V	327	51	15.6	Syn
		1614	A→G	538	E→E	399	109	27.32	Syn
		2265	G→A	755	R→R	880	34	3.86	Syn

alt. (alternative), ref. (reference), nsy(Nonsynonymous) and syn (Synonymous).

**Figure 3:**
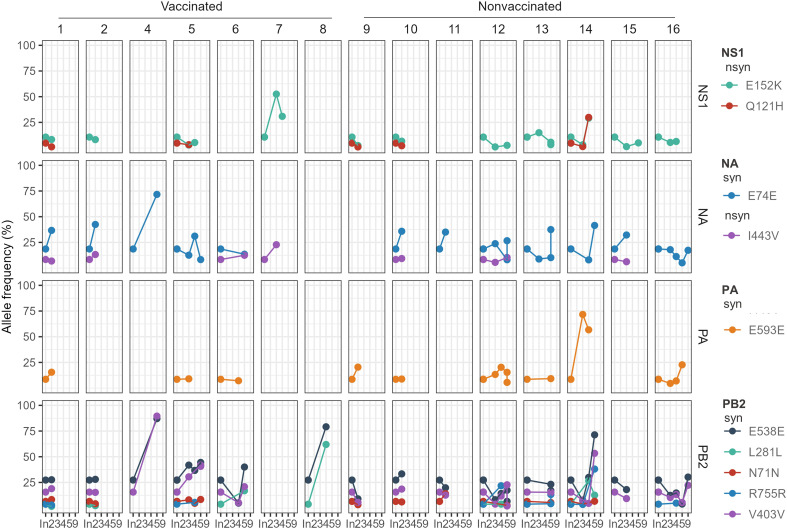
Allele frequencies evolution of H1N2 inoculum SNVs over time in pig sequenced samples. The SNV allele frequency percentage is represented in the y-axis. The time is represented in the x-axis, where “In.” is the day of the inoculation, and 2, 3, 4, 5, and 9 are the days post-inoculation. Each animal and genomic segment are represented in columns and rows, respectively. Dots show SNV detection plotted in different colors, while lines join the same mutation detected at different time points in the same animal. *nsyn (Nonsynonymous)* and *syn (Synonymous)*.

Most of these SNVs substitutions were later detected in sequenced samples collected from both, vaccinated and nonvaccinated animals (**Figure 3**). All these substitutions tended to reduce their allelic representation over time or were not even detected again in most animal sequenced samples, with a few exceptions. Nonsynonymous substitution in the NS1 gene only increased its allele frequency in pig No. 14, exceeding in both cases the 25% of allele frequency. Regarding synonymous substitutions, the E74E (NA) allele frequency increased in many of the samples, especially in the vaccinated animal No. 7. In the E593E (PA) substitution, the allele frequency only increased in pig No. 14. By last, in the PB2 segment, the E538E substitution increased its allelic frequency in many of the samples later sequenced, especially in animals No. 4, 8, and 14. Likewise, the frequency of the V403V substitution also tends to increase, particularly in animals 4 and 14. Finally, the L281L substitution tended to decrease its allelic frequency, except in animals No. 6 and 8 (**Figure 3**).

### 3.5 *De novo* SNVs identification; synonymous and nonsynonymous proportion and allocation along SIAV genome

**Figure 4:**
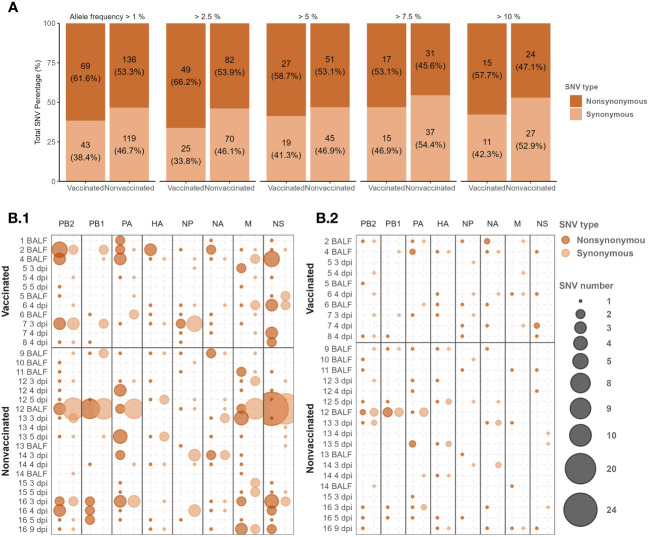
*De novo* H1N2 SNVs detected in samples collected from Vaccinated and nonvaccinated animals after challenge. **(A)** Synonymous and non-synonymous SNVs proportion bars with allele frequencies greater or equal to 2.5, 5, 7.5, and 10 per group. The total number of variants found and the percentage that it represents by type of variant, group, and allele frequency studied are indicated. **(B)** H1N2 *de novo* SNV allocation bubble plot representation with an allele frequency greater than 1 (B.1) and 5% (B.2). The bubble size proportionally represents the total number of SNVs found per genome segment (x-axis) and sequenced sample (y-axis). Synonymous and nonsynonymous substitutions are represented in light and dark orange, respectively.

In the present study, a total of 367 *de novo* SNV were detected, 112 in samples collected from vaccinated animals and 255 in samples collected from nonvaccinated ones (**Figure 4A**). Considering the number of sequences obtained per group, on average 9.33 substitutions per sample were produced in vaccinated animals, while 12.75 were produced in nonvaccinated ones, being this difference not significant (*t-test*).

In relation to the proportion of synonymous and nonsynonymous *de novo* SNVs counted per group, the nonsynonymous SNVs were proportionally more abundant. Interestingly, in vaccinated animals, the proportion of nonsynonymous substitutions was always higher than in nonvaccinated animals along all allelic frequencies analyzed, although significant differences were found (*chi-squared* test) (**Figure 4A**).

*De novo* SNVs variants were allocated along all SIAV genome segments in sequenced samples from both experimental groups (**Figure 4B**; **Supplementary T** **able S3**). In samples collected from nonvaccinated animals, most of the *de novo* nonsynonymous SNVs with an allele frequency greater than 1 were found in the polymerases (PB2, PB1, and PA), M, and NS segments. Regarding these types of SNVs in nonvaccinated pigs, 9, 11, and 10 out of 17, 17, and 20 substitutions exceeded an allelic frequency of 5% in PB2, PB1, and PA, respectively. Notably, only 4 out of the 21 nonsynonymous substitutions found in the M segment exceed the 5% of allele frequency. The largest number of nonsynonymous SNVs counted in nonvaccinated animals was found in the NS segment, although only 3 out of 39 SNVs were detected with an allelic frequency greater than 5%. On the contrary, the lowest number of mutations per segment in this group of animals was found in the NP segment with only 5 nonsynonymous SNVs (**Figure 4B**; **Supplementary Ta** **ble S3**).

On the other hand, in vaccinated animals, the highest number of nonsynonymous substitutions (n=17) with an allele frequency greater than 1 was also found in the NS segment, and 5 out of them exceeded 5%. In this group of animals, most of the nonsynonymous substitutions in polymerase segments were found in PB2. In the PA, only 2 substitutions were found, being the lowest number of nonsynonymous SNVs found. As in nonvaccinated animals, most substitutions found in polymerase segments did not exceed 5% allelic representation. In NP and M segments, 7 and 6 nonsynonymous substitutions were found, reaching 5% of allele frequency 4 and 1 of them, respectively.

Regarding nonsynonymous SNVs detected in the surface glycoproteins HA, and NA, according to the number of sequences obtained, proportionally more substitutions were found in vaccinated pigs. In the HA segment, with an allelic frequency of 1%, 7 substitutions were found in each group, and 4 of them exceeded 5% allelic representation in vaccinated animals while all did so in nonvaccinated ones. In the NA, 10 substitutions were found in nonvaccinated pigs while 7 were found in the vaccinated group. Considering the 5% of allele frequency, more substitutions were found in vaccinated animals with 5 substitutions found, meanwhile 4 were found in nonvaccinated group.

### 3.6 *De novo* nonsynonymous SNV with an allele frequency greater than 5% allocation on protein domains

**Figure 5:**
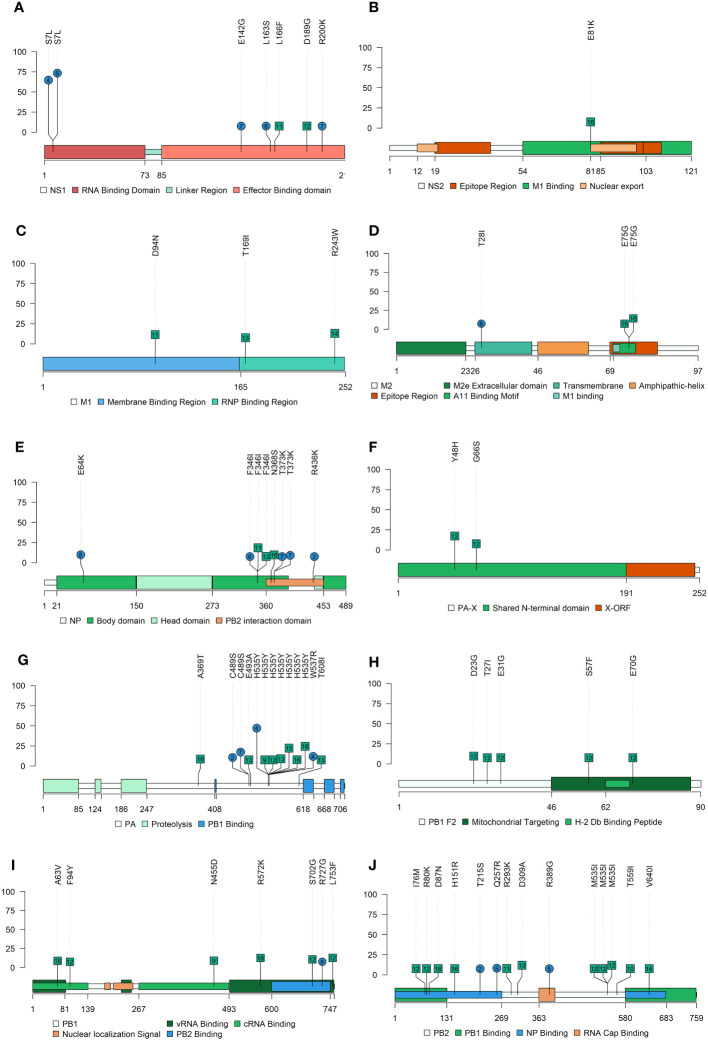
Nonsynonymous SNV allocation on SIAV proteins lolliplot representation. Substitutions found in NS1 **(A)**, NS2 **(B)**, M1 **(C)**, M2 **(D)**, NP **(E)**, PA **(F)**, PA-X **(G)**, PB2-F2 **(H)**, PB1 **(I)** and PB2 **(J)** proteins with an allele frequency greater than 5%. In ordinate axis, the allele frequency of each substitution is indicated. Substitutions noted in blue circles and green squares show substitutions found in vaccinated and nonvaccinated animals, respectively. Number inside each shape indicates the pig ID of each reported substitution. Figure legends indicate the most important domains of each protein.

**Figure 6:**
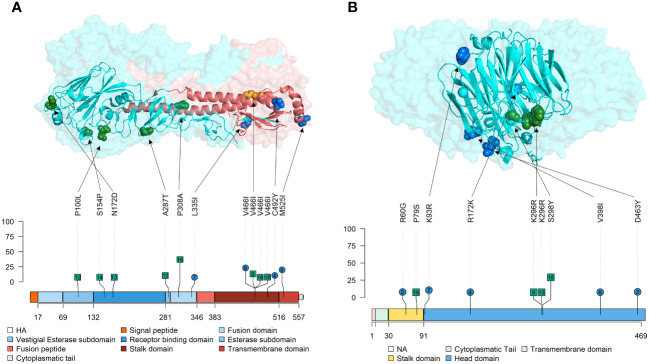
Location of nonsynonymous SNVs found with an allele frequency greater than 5% on Tri-Dimensional and Lolliplot surface SIAV glycoproteins HA and NA representation. **(A)** HA trimer (PDB accession no. 3LZG (Xu et al., 2010)). HA1 domains are represented in blue, while HA2 ones are represented in red colors. **(B)** NA tetramer (PDB accession no. 4B7Q (van der Vries et al., 2012)). In lolliplot representation, substitutions found in vaccinated and nonvaccinated animals are represented in blue circles and green squares, respectively. The numbers within each mutation marked on the lolliplot show the animal in which it was detected. The numbers below each protein representation indicate the amino acid boundaries between the different domains. The allele frequency at which each substitution was reported is indicated in the ordinate axis.

In the present study, the following *de novo* SNVs substitutions with an allele frequency were detected in vaccinated animals in each SIAV H1N2 protein; in PB2 (T215S, Q257R, and R389G), in PB1 (R727G), PA (C489S, H535Y, and W537R), HA (L335I, V466I, C492Y, and M525I), in NP(E64K, F346I, T373K, and R436K), NA (R60G, K93R, R172K, V398I, and D463Y), M2(T28I) and NS1(S7L, E142G, L163S, and R200K) (**Figures 5**, **6**). Meanwhile, in nonvaccinated animals nonsynonymous substitutions which allele frequency exceed 5% were reported in PB2 (I76M, R80K, D87N, H151R, R293K, D309A, M535I, T559I, and V640I), in PB1 (A63V, F94Y, N455D, R572K, S702G, and L753F), in PB1-F2 (D23G, T27I, E31G, S57F, and E70G), PA-X (Y48H, and G66S), PA (A369T, E493A, H535Y, and T608I), HA (P100L, S154P, N172D, A278T, P308A, and V466I), NP(F346I, and N368S), NA (P79S, K296R, and S298Y), matrix protein (M1) (D94N, T169I, and R243W), ion channel (M2) (E75G), NS2 (E81K) and NS1(L166F and D189G). All the substitutions found in this study with an allele frequency greater than 1% were noted in the **Supplementary Table S4**.

### 3.7 Nucleotide diversity

**Figure 7:**
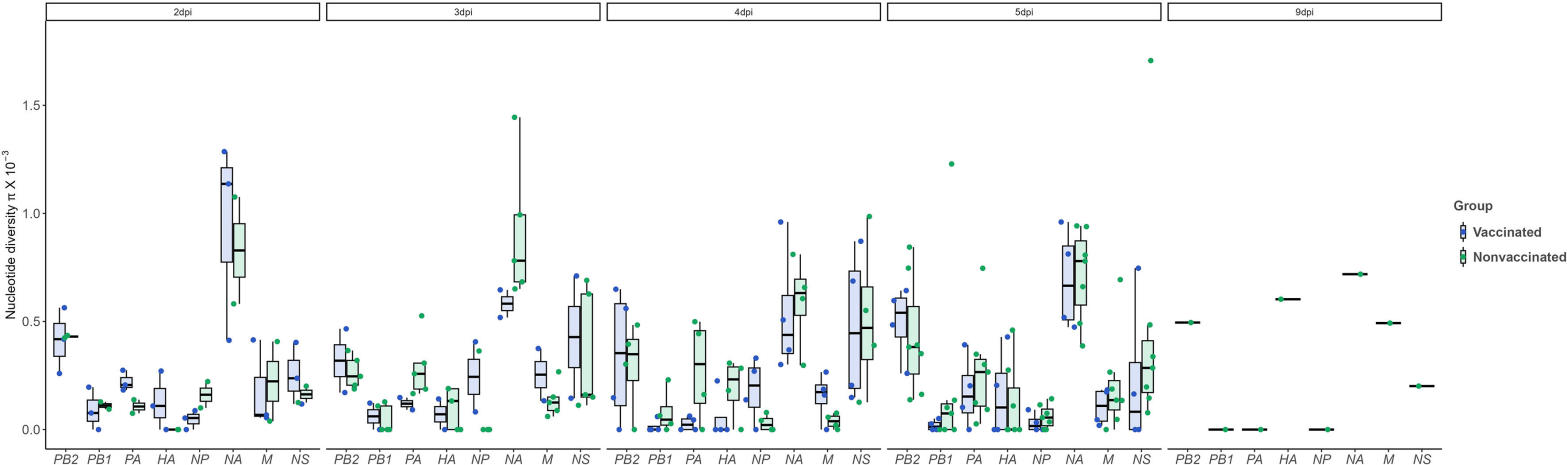
Nucleotide diversity (π) over time and per genomic segment. Boxplots indicate means, lower and upper quartile, and standard deviation. The π represented in blue and green colors correspond to samples collected from vaccinated and nonvaccinated animals, respectively. No significant differences were found. ANOVA test was used for statistical analysis.

Regarding nucleotide diversity, it was high for NA at day 2 and 3 dpi, while the trend at days 4 and 5 dpi was decreasing in both experimental groups, although significant differences were not found (**Figure 7**).

## 4 Discussion

The high capacity of mutation and adaptability of IAVs, the wide host range, and their interspecies transmission, make of IAVs a constant threat to both, human and animal health (Mostafa et al., 2018). Due to the important role of swine in human-to-avian IAVs adaptation, the study of SIAVs evolution might be of great value to understand their genetic diversity and interaction with the host to avoid new strain emergence. Nowadays, vaccine application is used in swine population to reduce the SIAV circulation and mitigate the disease (Reeth et al., 2016; Salvesen and Whitelaw, 2021). However, genetic differences between SIAV strains used in vaccine formulation and current circulating strains, make that the immunity generated by vaccine is not complete, allowing virus replication and evolution. Previous studies have shown different evolution patterns on H1N1 and H3N2 SIAV subtypes, that may be caused by different host immune pressures (López-Valiñas et al., 2021; López-Valiñas et al., 2022; López-Valiñas et al., 2023). Nevertheless, the effect of vaccine application on H1N2 SIAV evolution through NGS technology is poorly studied. The study of the evolution of this subtype could provide an enriching point of view about virus evolution, in addition to the fact that it is one of the main currently circulating SIAV in swine population (Simon et al., 2014; Ma, 2020), and one of the subtypes from which the pandemic A(H1N1)pdm virus arose (Garten et al., 2009). Hence, the present work focuses on the study of the evolutionary dynamics of SIAV *human-like* H1N2 subtype under the effect of the most widely used vaccine in the field.

Herein, a trivalent SIAV vaccine was applied to one group of animals. At day of the challenge, the antibody titters against SIAV NP were statistically higher in vaccinated animals. Regarding SIAV detection, it was detected in both experimental groups after the challenge. The viral loads detected in vaccinated animals were lower in nasal swab, BALF, lung, and NT in comparison with nonvaccinated ones. Moreover, the number of samples that tested RT-PCR positive were also lower in vaccinated pigs. After challenge, rectal temperatures were lower in vaccinated animals, increasing the temperature significantly in animals that did not receive the vaccine. In relation to pathological findings, the same pattern was found, with vaccinated animals showing less severe and extenSIAVe lesions. Hence, in the present study, although limited, the effect of the vaccine in viral shedding and clinical signs reduction was proved. However, viral replication was not prevented by vaccination, facilitating the virus to acquire novel nucleotide variants, as previously found (Murcia et al., 2012; López-Valiñas et al., 2021; López-Valiñas et al., 2022; López-Valiñas et al., 2023).

In the present work, 33 SIAV whole genome sequences were analyzed by NGS, including the H1N2 inoculum, 12 samples collected from vaccinated pigs, and 20 collected from nonvaccinated ones. Regarding the SNVs detected in the inoculum, a total of 10 SNVs were detected, 3 of them nonsynonymous ones (E152K and Q229H in NS1, and I443V in NA). All substitutions were also later detected in samples collected from both experimental groups, although their allelic frequencies in general decreased in comparison with the initial allelic frequency of the inoculum, or even they were no longer detected. Therefore, these mutations did not imply a benefit on viral fitness, so they were lost over time. Considering that virus was grown on MCDK cells before challenge, the nucleotide diversity loss of the inoculum could be explained by the virus readaptation of their natural host after a bottleneck effect (Sobel Leonard et al., 2016; Cortey et al., 2018).

In the present study, the rapid evolutionary capacity and plasticity of SIAV H1N2 under vaccination and nonvaccinated scenarios is again proved, as 367 substitutions were reported from sequences of all studied pigs, further supporting previous results with other SIAV strains (Murcia et al., 2012; Diaz et al., 2013; Diaz et al., 2015; López-Valiñas et al., 2021; López-Valiñas et al., 2022).

The nonsynonymous and synonymous *de novo* SNVs proportions, could provide an idea about which is the main force that is driving the virus evolution under different scenarios. In this work, the proportion of nonsynonymous SNVs was greater than synonymous ones in both experimental groups. Notably, the proportion of nonsynonymous substitutions was always higher in vaccinated animals, maintaining this dynamic throughout all the variant allelic frequencies analyzed. This could be indicating that the immune pressure of the vaccinated pigs was additionally acting as positive selection force in the evolution of the virus (Fitch et al., 1991; Li et al., 2011), as previously reported for H1N1 and H3N2 SIAV subtypes (López-Valiñas et al., 2021; López-Valiñas et al., 2022; López-Valiñas et al., 2023). On the other hand, this proportion was around 50% for sequences from nonvaccinated animals suggesting that a neutral selection could be acting in this scenario (Frost et al., 2018). Thus, the genome of SIAV is diversifying in both scenarios (vaccination versus non-vaccination), acquiring synonymous substitutions that could play a neutral role in the evolution of the virus, and nonsynonymous substitutions that could have negative or positive role in its evolution.

According to the quasispecies evolution theory, those mutations that poses a beneficial for virus fitness subsequently appear in the viral progeny, being naturally selected and, consequently, its allele frequency increases on virus population over time (Domingo and Perales, 2019). Herein, 142 out of 367 (38.7%) *de novo* SNVs were reported with an allele frequency greater than 5%. Therefore, most of the substitutions generated in the SIAV genome during the trial could have a negative impact on viral fitness or, alternatively, more time is required for one variant to become more dominant. For instance, a total of 135 nonsynonymous SNVs were found in PB2, PB1, PA, and NS segments, although only 45 reached the 5% of allele frequency. However, those substitutions with an allele frequency greater than 5% found in vaccinated animals could play an important role in the adaptation of the viral quasispecies, posing an evolutionary advantage against host immune pressure. No substitutions were found in NS2, M1, PA-X and PB1-F2 proteins in vaccinated animals. Furthermore, in the polymerase segments (PB2, PB1, PA), the number of nonsynonymous substitutions found were lower in samples from vaccinated animals, being only one detected in PA protein. Substitutions found in polymerase proteins could increase the IAV polymerase activity, the virulence, and interspecific host jump, according to previous literature (Subbarao et al., 1993; Song et al., 2015; Yu et al., 2015; Cai et al., 2020).

In the NS1 protein, 6 *de novo* SNVs were found in vaccinated animals meanwhile only 2 were reported from nonvaccinated ones. Substitution S7L in the RNA Binding Domain was simultaneously reported with a high allele frequency in two vaccinated animals (No. 4 and 6). The remaining SNVs detected in vaccinated animals, E142G, L163S, and R200K, were found in the Effector Binding Domain of the protein. According to previous literature, substitutions on this domain may be involved in the regulation of the interferon (IFN) type I and in the viral mRNA translation enhancement, causing as a result the increase of viral replication and virulence (Nemeroff et al., 1998; Aragón et al., 2000; Jia et al., 2010; Thepmalee et al., 2013; Nogales et al., 2017). In a previous similar study with the SIAV H1N1 subtype, substitutions in the NS1 protein were only reported from vaccinated animals (López-Valiñas et al., 2021). Furthermore, the substitution L163S (as well as the G161E substitution reported in the previous mentioned study) was allocated in a region equivalent to the human leukocyte antigen B7 (HLA-B7) binding motif (Gianfrani et al., 2000) that could be related with swine leukocyte antigen B7 (SLA-B7). Hence, herein it could be again hypothesized that the plasticity of the NS1 could help virus adaptability under immune pressure. In relation with the NP, 7 substitutions were found, 4 of which in samples from vaccinated animals. In previous studies, substitutions were also found in vaccinated animals previously challenged with the H1N1 subtype (López-Valiñas et al., 2021). However, substitutions were not reported in the H3N2 subtype (López-Valiñas et al., 2022; López-Valiñas et al., 2023). Hence, it could be inferred that the plasticity of NP is different depending on the subtype, being greater in H1N1 and H1N2 ones, while H3N2 NP seems to be more conserved. According to previous studies, the existence of highly conserved NP sequences between different subtypes, would open the avenue for a good candidate for IAV universal vaccine development (Zheng et al., 2014; Hu et al., 2017; Withanage et al., 2022). Therefore, these substitutions generated in H1N1, and H1N2 NP are probably not able to establish themselves in the SIAV genome over time, ultimately resulting in its loss.

The nonsynonymous SNVs found in the surface glycoproteins, HA, and NA, may be changing the antigenicity of the virus, as these proteins are the main target for the host to generate neutralizing antibodies after SIAV exposure by natural infection or vaccination (Li et al., 2011; Eichelberger and Wan, 2014; Ma, 2020). Thus, both proteins are under positive selection forces, acquiring more genetic diversification (Li et al., 2011; Krammer, 2019). The percentages of nucleotide identity between the H1N2 vaccine subtype and the virus challenge are 93.19% for HA and 90,14% for NA, which are similar to other isolates. The HA is the protein responsible for the virus sialic acid cell attachment and subsequent entry (Russell, 2021). Herein, 4 *de novo* substitutions with an allele frequency greater than 5% were detected in vaccinated animals while 6 were detected in nonvaccinated ones. In vaccinated animals, substitutions L335I in the fusion domain, V466I and C492Y in the stalk domain, and M525I in the transmembrane domain, were allocated in the HA with an allele frequency greater than 5%. In previous studies, relevant substitutions in quasispecies collected from vaccinated animals were found in the stalk domain of the protein (López-Valiñas et al., 2021; López-Valiñas et al., 2022; López-Valiñas et al., 2023). According to previous literature, the stalk region of the protein evolves independently from immune pressure (Kirkpatrick et al., 2018), although our results suggest that this domain could be evolving under immune pressure. Furthermore, substitutions in the transmembrane domain of the HA were also reported in a similar study with the H1N1 subtype (López-Valiñas et al., 2021). Substitutions in this domain could affect its interaction with surrounding bilayer lipids and, as a SIAV result of the protein differently fold, its antigenic exposure can be modified (Kubiszewski-Jakubiak and Worch, 2020).

Regarding NA, this protein is responsible for releasing the viral progeny at the end of the lytic viral cycle (McAuley et al., 2019). In vaccinated animals, a total of 5 nonsynonymous substitutions were reported with an allele frequency greater than 5%; the R60G mutation is in the stalk domain, while the remaining K93R, R172K, V398I, and D463Y are allocated in the head domain of the protein, in the most exposed part. Deletions in stalk domine have been related with a major viral shedding and a major proportion of animals remaining potentially infectious (Deblanc et al., 2020), thus, R60G can be involved in vaccine escape. Substitution D463Y is allocated in a previous described epitope N2 region L’463–468 (Ge et al., 2022). Nonsynonymous substitutions were also found in nonvaccinated animals, K296R and S298Y, both in the head domain. Proportionally to the number of sequences obtained per group, more substitutions were found in the vaccinated animals. Further analyses with reverse genetic technology would be required (Li et al., 2021) to determine the effect of each substitution herein found on surface glycoproteins. The nucleotide diversity analysis disclosed a higher value for NA segment in both groups, particularly at 2 and 3dpi, although a high value was presented in the inoculum. This is in contrast with the values found for H1N2 subtype where HA presented a higher nucleotide diversity (López-Valiñas et al., 2022). This opposite behaviour of the HA from H1N2 subtype and the HA from H1N2 reveals how reassortments can modify the dynamic of the evolution of SIAV.

Although the function of *de novo* SNVs in HA should be further determined to shed light on viral pathogenesis and protection against this virus, the obtained results highlight the wide evolution and plasticity capacity of IAVs, assuming an evolutionary success due to the quick adaptation of viruses to different environments. Such results further support a continuous IAV circulation through ecosystems and evolution, posing a threat to global health. Hence, the improvement of biosecurity conditions on farms, sustainable meat consumption to contribute to a more rational livestock production, the increase in genetic and surveillance studies, the expansion of current vaccines use against IAV and the development of universal vaccines are necessary measures to minimize virus circulation and, therefore, reduce its endemic and pandemic risks.

### 4.1 Permission to reuse and copyright

Permission must be obtained for use of copyrighted material from other sources (including the web). Please note that it is compulsory to follow figure instructions.

## Data availability statement

The datasets presented in this study can be found in online repositories. The names of the repository and accession number can be found below: https://www.ncbi.nlm.nih.gov/genbank/, PRJNA994299.

## Ethics statement

The animal study was approved by Animal ethics committee from the Generalitat de Catalunya (project number 10442). The study was conducted in accordance with the local legislation and institutional requirements.

## Author contributions

ÁL-V: Methodology, Software, Writing – original draft. MV: Methodology, Writing – review & editing. MP: Methodology, Writing – review & editing. AD: Funding acquisition, Writing – review & editing. CC: Methodology, Writing – review & editing. LG: Methodology, Writing – review & editing. JS: Methodology, Writing – review & editing. JIN: Methodology, Writing – review & editing, Conceptualization, Funding acquisition.
